# Estimating an Individual’s Probability of Revision Surgery After Knee Replacement: A Comparison of Modeling Approaches Using a National Data Set

**DOI:** 10.1093/aje/kwy121

**Published:** 2018-06-11

**Authors:** Parham Aram, Lea Trela-Larsen, Adrian Sayers, Andrew F Hills, Ashley W Blom, Eugene V McCloskey, Visakan Kadirkamanathan, Jeremy M Wilkinson

**Affiliations:** 1 Department of Automatic Control and Systems Engineering, University of Sheffield, Sheffield, United Kingdom; 2 School of Clinical Sciences, University of Bristol, Bristol, United Kingdom; 3 School of Social and Community Medicine, University of Bristol, Bristol, United Kingdom; 4 Department of Oncology and Metabolism, University of Sheffield, Sheffield, United Kingdom; 5 Centre for Integrated Research in Musculoskeletal Ageing, University of Sheffield, Sheffield, United Kingdom

**Keywords:** calibration, discrimination, flexible parametric survival model, knee replacement, parametric survival model, random survival forest, revision surgery, time-to-event analysis

## Abstract

Tools that provide personalized risk prediction of outcomes after surgical procedures help patients make preference-based decisions among the available treatment options. However, it is unclear which modeling approach provides the most accurate risk estimation. We constructed and compared several parametric and nonparametric models for predicting prosthesis survivorship after knee replacement surgery for osteoarthritis. We used 430,455 patient-procedure episodes between April 2003 and September 2015 from the National Joint Registry for England, Wales, Northern Ireland, and the Isle of Man. The flexible parametric survival and random survival forest models most accurately captured the observed probability of remaining event-free. The concordance index for the flexible parametric model was the highest (0.705, 95% confidence interval (CI): 0.702, 0.707) for total knee replacement and was 0.639 (95% CI: 0.634, 0.643) for unicondylar knee replacement and 0.589 (95% CI: 0.586, 0.592) for patellofemoral replacement. The observed-to-predicted ratios for both the flexible parametric and the random survival forest approaches indicated that models tended to underestimate the risks for most risk groups. Our results show that the flexible parametric model has a better overall performance compared with other tested parametric methods and has better discrimination compared with the random survival forest approach.

Shared decision-making between patient and doctor is fundamental to good clinical practice (1, 2) and improves patient knowledge about medical treatments and their associated benefits and risks (3). Decision aids fill the gap between population-level data and its application to the patient’s individual circumstances to better inform patients making choices about health-care interventions (4–6). The use of decision aids in controlled settings enhances patient participation in the process, improves their knowledge and satisfaction, and reduces decisional conflict (1, 7–9). Patient engagement through shared decision-making reduces inequalities in health between patient groups and benefits health-care economies through improved clinical outcomes and better resource utilization (10).

Osteoarthritis is the most prevalent musculoskeletal disease and is a leading cause of chronic pain and disability worldwide (11–13). In the United Kingdom alone, 9 million people currently seek treatment for osteoarthritis with a total indirect cost to the economy of £14.8 billion (approximately $19.6 billion) per annum (14, 15). Each year almost 100,000 individuals undergo knee replacement surgery in England and Wales (16), with a direct cost of £546 million (approximately $722 million) for the inpatient stay alone (14). The National Joint Registry for England, Wales, Northern Ireland, and the Isle of Man (NJR) (http://www-new.njrcentre.org.uk/) was established in 2003 to collect audit data on all total hip and knee replacement surgery in these regions, for which it has a completeness rate of 97% (17).

Evidence-based decision-making in the setting of joint replacement surgery, where such decisions are preference-sensitive (6, 18), enable the patient to arrive at an informed choice among several alternative treatments (5). The development of a personalized decision aid in this setting requires the generation of a time-to-event model that incorporates individual characteristics, prosthesis choice, and other fixed and modifiable risk factors. The choice of such models is potentially large, including semiparametric Cox models, parametric survival models, flexible parametric survival models (FPMs), and random survival forests (RSFs). These models can be adapted to provide an estimate of the absolute risk of the outcome of interest for each individual. We used the NJR data set to assess the performance of these methods for individual prediction of the risk of prosthesis revision over an 8-year interval after knee replacement.

## METHODS

### Study population

Our base data set included 787,106 knee replacements carried out in England and Wales between April 2003 and September 2015. We excluded procedures for which osteoarthritis was not the only indication for surgery (29,918), the patient’s body mass index (BMI, calculated as height (h)/weight (m)^2^) was below 15 or above 55 (2,485), the patient was younger than 30 years or older than 100 years (262), or the American Society of Anesthesiologists grade was 4 or 5 (2,782), indicating severe comorbidities. We conducted a complete-case analysis and excluded procedures with missing data on any of the study covariates, namely: BMI (316,828 missing), knee replacement procedures (10,648 missing), and chemical and mechanical thromboprophylaxis (1,589 missing). This resulted in 430,455 cases with complete information.

Separate models were constructed for each of the procedures being considered—total knee replacement (TKR), unicondylar knee replacement (UKR), or patellofemoral replacement (PFR)—due to differences in survival performance characteristics of the different prosthesis categories (19).

### Outcome and covariates

The outcome of interest in our time-to-event models was time to first revision surgery. We linked primary knee replacement procedures to revision procedures recorded in the NJR using a unique patient identifier and side (left or right knee). Patient death with a nonrevised prosthesis was considered to be a censoring event. Analysis covariates included age, BMI, sex, American Society of Anesthesiologists grade, chemical and mechanical thromboprophylaxis, and operation type (unilateral/same-day bilateral), based on their known association with prosthesis revision (19–21). The revision of each side of both simultaneous and sequential bilateral procedures was considered independently, with separate times to event for each side. Sequential bilateral procedures performed on different dates were considered to be independent unilateral operations. Previous research has shown that ignoring the potential dependence between procedures in the same patient does not lead to bias (22).

### Modeling approaches

In standard parametric methods a distribution for time-to-event data is assumed where the unknown parameters are inferred using the maximum likelihood estimation. Here, we considered exponential, Weibull, and log-logistic distributions. The exponential distribution is defined by a single scale parameter and assumes a constant hazard over time. The Weibull distribution is a 2-parameter distribution with scale and shape parameters producing increasing (shape parameter >1) and decreasing (shape parameter <1) monotonic hazard functions (23). The Weibull and exponential models are proportional hazards models. The 2-parameter log-logistic model is a proportional odds model that can produce a decreasing monotonic (shape parameter ≤1) or unimodal (shape parameter >1) hazard function, depending on the shape parameter (24).

If the estimation of the time-to-event distribution itself is not required, the semiparametric Cox model can be used to estimate the effect of covariates on the baseline hazard function. The Cox model assumes proportional hazards and can be fitted by maximizing a partial likelihood function (25, 26).

The standard parametric models explained above place specific constraints on the shape of the hazard function. The FPM offers an alternative approach such that restrictions on the shape of the hazard function are relaxed (27). In this approach the baseline cumulative hazard or odds function is modeled as a flexible function of log time using restricted cubic splines. Restricted cubic splines are piecewise third-order polynomials that are smoothly joined together at break points or knots (28). The complexity of the baseline distribution is determined by the number and position of knots in the spline function. Optimal placement of knots is not essential; thus a simple centile-based approach can be adopted (28). The model is fitted with either a proportional hazards or odds assumption using maximum likelihood estimation.

The RSF algorithm (29) is a machine learning tool for modeling time-to-event data and is an extension of random forest classifiers and regressors introduced by Breiman (30). The RSF is a distribution-free method, and its tree-based architecture can take possible interaction effects into account through hierarchical splitting. The RSF approach also accounts for nonlinearity by dichotomizing continuous variables at split points (29). In RSF *B* bootstraps are drawn from the original data set, and each bootstrap sample is used as a root node to grow a survival tree. A subset of covariates is randomly selected at each node of the tree. The node is then split into 2 left and right daughter nodes using a covariate that gives the maximum survival difference between daughter nodes. This can be done through a measure of separation such as the log-rank test (31–33). For continuous covariates splits over all possible values are considered, and an optimal cutoff is then chosen. The tree is grown until each terminal node contains at least a prespecified number of unique cases. For every tree the cumulative hazard function for each terminal node can be calculated using the Nelson-Aalen estimator (34, 35). This gives a series of estimators that correspond to different terminal nodes that define the cumulative hazard function for the tree. The estimated tree’s hazard function for an individual is the Nelson-Aalen estimator for the individual’s terminal node, and an average cumulative hazard function is calculated across all trees in the random forest. It is recommended that between 64 and 128 trees be used to achieve a balance between model performance, processing time, and memory use (36).

### Overall model performance

We used the Akaike information criterion (AIC), a measure that compromises between goodness-of-fit and model complexity (37), to provide an overall measure of the performance of the parametric models. We also compared model predictions by averaging the time-to-event estimates for individuals at each time point and comparing with the population-based estimation (Kaplan-Meier).

### Model validation

We applied repeated *m*-fold cross-validation to measure the performance of candidate models’ overall predictive value, discrimination ability, and calibration (38). In *m*-fold cross-validation, the data set is randomly assigned into *m* partitions of approximately equal size. The model is then constructed *m* times using *m* − 1 of the partitions and tested on the remaining part of the data. The *m* test results are then averaged to compute an overall performance measure. This ensures that all available data is used for training and testing the models. In repeated cross-validation the above procedure is performed several times. This reduces the variation of the *m*-fold cross-validation due to the random partitioning (39) and also allows the computation of confidence intervals for performance measures.

Overall validation performance: We evaluated the overall performance of models using the time-dependent Brier score, a commonly used tool in clinical outcomes analysis (40). The Brier score is a proper score function that evaluates the accuracy of probabilistic forecasts, and is calculated as the weighted average of squared distances between the observed outcome and predicted probability of that outcome at fixed time points (41). The weights are introduced to incorporate information from censored data and calculated using a model for either marginal or conditional censoring distribution. Time-dependent Brier scores can be integrated over time to provide a summary measure of overall performance. The nearer the Brier score is to zero for a set of predictions, the better the predictions match the observed outcomes.

Discrimination: We evaluated the discrimination capability of our models using an extension of Harrell’s concordance index (*C* index) (42). The Harrell’s *C* index is the proportion of pairs of subjects in which the one with the shorter time to event is associated with a higher predicted risk. This ignores pairs where the shorter times to event are censored to produce a result that depends on the censoring distribution. This is addressed by introducing a weighted *C* index, where the weights are similar to that of the Brier score (43).

Calibration: The models were further validated using a calibration process. Calibration is used to test the agreement between the predicted risks and the observed risks for different risk groups. These risk groups can be formed by dividing the predicted risk into quantiles. The observed risk for each group can then be computed using Kaplan-Meier method within that risk group (44).

### Statistical analysis

We implemented different time-to-event models for each of the TKR, UKR, and PFR procedures with the same set of covariates. We performed a complete-case analysis assuming that data were missing at random, and used only cases with complete data on the covariates of interest. In parametric models a linear combination of the covariate vector is used to form the risk score. We also investigated nonlinear associations of age and BMI with the outcome using first-degree and second-degree fractional polynomials (45). The number of unknown parameters in the baseline hazard function depends on the chosen model: 1 for the exponential model and 2 for Weibull and log-logistic models. For the FPMs we used AIC values as guidance for selection of the scale, proportional hazards or odds, and the number of knots as proposed by Royston and Parmar (27). In the RSF approach each random forest was computed using 100 bootstraps samples and the log-rank splitting rule.

The parametric models, estimated by maximum likelihood, were compared using AIC values. We also compared average (over individuals) prediction of each model with Kaplan-Meier estimates.

We then selected the models that could capture the overall survival pattern and further evaluated them using 50 repeats of 5-fold cross-validation by comparing the Brier score, *C* index, and calibration plot. We also performed our evaluation using 50 repeats of stratified 5-fold cross-validation (46) where each fold contained the same proportion of revised and unrevised cases as in the original data.

The statistical analyses were carried out using R (R Foundation for Statistical Computing, Vienna, Austria) (packages: randomForestSRC (47), survival (48, 49), flexsurv (50) and pec (51)).

## RESULTS

Baseline characteristics of the complete data set are given in Table 1.

**Table 1.** Baseline Characteristics of the Patient-Procedure Episodes in the Complete Data Set From the National Joint Registry for England, Wales, Northern Ireland, and the Isle of Man, 2003–2015

Characteristic	TKR			UKR			PFR		
	No.	%	PTIR	No.	%	PTIR	No.	%	PTIR
Outcome									
Unrevised	381,322	98.4		36,009	95.5		4,937	93.1	
Revised	6,137	1.6		1,684	4.5		366	6.9	
Age, years	70.2 (9.1)^a^		0.45	64.0 (9.7)^a^		1.25	59.6 (11.4)^a^		1.90
BMI^b^	70.2 (9.1)^a^		0.45	30.1 (5.0)^a^		1.25	29.5 (5.3)^a^		1.90
Sex									
Female	221,178	57.1	0.41	17,542	46.5	1.30	4,148	78.2	1.73
Male	166,281	42.9	0.50	20,151	53.5	1.21	1,155	21.8	2.53
ASA physical status									
P1 (healthy patient)	39,075	10.1	0.49	8,179	21.7	1.32	1,378	26	1.80
P2 (mild systemic disease)	286,693	74.0	0.44	26,432	70.1	1.22	3,503	66.1	1.95
P3 (severe systemic disease)	61,691	15.9	0.49	3,082	8.2	1.37	422	8.0	1.82
Chemical prophylaxis									
None	23,418	6.0	0.43	2,863	7.6	1.31	407	7.7	2.18
Aspirin only	27,996	7.2	0.42	4,407	11.7	1.16	745	14.0	1.63
LMWH ± aspirin	248,124	64.0	0.45	21,518	57.1	1.29	2,949	55.6	2.05
Other/other combinations	87,921	22.7	0.47	8,905	23.6	1.19	1,202	22.7	1.52
Mechanical prophylaxis^c^									
None	23,418	6.0	0.47	1,273	3.4	1.68	249	4.7	2.75
Active	84,589	21.8	0.46	8,476	22.5	1.17	1,234	23.3	1.45
Passive	125,239	32.3	0.44	11,820	31.4	1.22	1,488	28.1	2.31
Both	148,761	38.4	0.45	15,775	41.9	1.27	2,231	42.1	1.76
Other/other combinations	5,452	1.4	0.35	349	0.9	1.63	101	1.9	1.14
Operation type									
Unilateral	381,650	98.5	0.45	35,542	94.3	1.29	4,791	90.3	2.02
Simultaneous bilateral	5,809	1.5	0.31	2,151	5.7	0.75	512	9.7	0.80

Abbreviations: ASA, American Society of Anesthesiologists; BMI, body mass index; LMWH, low molecular-weight heparin; PFR, patellofemoral replacement; PTIR, patient-time incident rate; SD, standard deviation; TKR, total knee replacement; UKR, unicondylar knee replacement.

^a^ Values are expressed as mean (SD).

^b^ Weight (kg)/height (m)^2^.

^c^ In mechanical prophylaxis, “active” includes foot pump and calf compression whereas “passive” is thromboembolic disease (TED) stockings.

For the FPMs we used proportional hazards scale with 3 interior knots for TKR and UKR models and 1 interior knot for the PFR model. For TKR and UKR models the internal knots were placed at quartiles of the log uncensored survival times, which resulted in 5 parameters in the baseline hazard function. For the PFR model, the internal knot was placed at the median of the log uncensored survival times, giving 3 parameters in the baseline hazard function. Partial dependence analysis based on predictions from RSF (52) suggested nonlinear associations between age and BMI and the outcome. We further analyzed these associations with the FPM using fractional polynomial fitting (45). The results are shown in Web Table 1 (available at https://academic.oup.com/aje), where only powers with the largest deviance differences are reported. The results show that the reduction in deviance is not significant (*P* ≥ 0.05) compared with the case where untransformed variables were used.

In RSF, age and BMI were always selected for splits, but other results for other variables were less stable. Mechanical prophylaxis, chemical prophylaxis, and American Society of Anesthesiologists grade were moderately selected for splitting nodes, while sex and operation type were selected in a small fraction of the resamples.

The 3 parametric proportional hazards models, log-logistic model, and the semiparametric Cox model for TKR are presented in Table 2 (UKR and PFR are shown in Web Tables 2 and 3). The hazard ratios from the parametric proportional-hazards models were in close agreement with the Cox semiparametric model. Note, the hazard ratio estimates of the FPM approach are closer to that of the Cox model compared with other proportional hazards models. This is expected given that the Cox model and the FPM should give unbiased hazard ratios whereas the hazard ratios conditional on a specific parametric model could be biased if the distribution is misspecified. The odds ratios of the log-logistic model also showed a consistent behavior with respect to hazard ratios.

**Table 2.** Parametric and Semiparametric Cox Models of Prosthesis Survivorship for Total Knee Replacement Using Data From the National Joint Registry for England, Wales, Northern Ireland, and the Isle of Man, 2003–2015

Characteristic	Exponential Model		Weibull Model		FPM		Cox Model		Log-Logistic Model	
	HR	95% CI	HR	95% CI	HR	95% CI	HR	95% CI	OR	95% CI
Age, years	0.955	0.953, 0.958	0.953	0.950, 0.956	0.955	0.953,0.958	0.955	0.953, 0.958	0.953	0.950, 0.956
BMI^a^	1.009	1.004, 1.014	1.009	1.004, 1.014	1.008	1.003, 1.013	1.008	1.003, 1.013	1.009	1.004, 1.014
Sex										
Female	1.000	Referent	1.000	Referent	1.000	Referent	1.000	Referent	1.000	Referent
Male	1.211	1.151, 1.274	1.222	1.158, 1.289	1.207	1.148, 1.270	1.207	1.148, 1.270	1.224	1.160, 1.291
ASA physical status										
P2 (mild systemic disease)	1.000	Referent	1.000	Referent	1.000	Referent	1.000	Referent	1.000	Referent
P1 (healthy patient)	0.925	0.854, 1.003	0.924	0.849, 1.005	0.932	0.860, 1.010	0.932	0.860, 1.010	0.923	0.848, 1.005
P3 (severe systemic disease)	1.229	1.146, 1.319	1.240	1.152, 1.335	1.225	1.142, 1.314	1.224	1.141, 1.312	1.242	1.154, 1.338
Chemical prophylaxis										
LMWH ± aspirin	1.000	Referent	1.000	Referent	1.000	Referent	1.000	Referent	1.000	Referent
Aspirin only	0.931	0.851, 1.018	0.938	0.854, 1.030	0.979	0.895, 1.071	0.980	0.896, 1.072	0.939	0.855, 1.033
None	0.969	0.884, 1.063	0.982	0.891, 1.081	1.028	0.938, 1.128	1.029	0.938, 1.128	0.983	0.891, 1.083
Other/other combinations	1.034	0.966, 1.106	1.020	0.950, 1.096	0.969	0.905, 1.037	0.963	0.900, 1.030	1.018	0.948, 1.094
Mechanical prophylaxis^b^										
Both	1.000	Referent	1.000	Referent	1.000	Referent	1.000	Referent	1.000	Referent
Active	0.993	0.927, 1.06	0.991	0.921, 1.065	0.982	0.917, 1.053	0.982	0.917, 1.053	0.990	0.921, 1.065
Passive	0.973	0.916, 1.034	0.974	0.914, 1.038	0.979	0.921, 1.041	0.981	0.923, 1.042	0.974	0.914, 1.039
None	1.017	0.924, 1.120	1.030	0.931, 1.139	1.068	0.938, 1.128	1.068	0.969, 1.176	1.031	0.931, 1.142
Other/other combinations	0.784	0.613, 1.004	0.776	0.600, 1.006	0.797	0.623, 1.020	0.797	0.622, 1.020	0.774	0.598, 1.004
Operation type										
Unilateral	1.000	Referent	1.000	Referent	1.000	Referent	1.000	Referent	1.000	Referent
Simultaneous bilateral	0.602	0.480, 0.756	0.589	0.464, 0.748	0.610	0.486, 0.765	0.609	0.486, 0.764	0.587	0.463, 0.746

Abbreviations: ASA, American Society of Anesthesiologists; BMI, body mass index; CI, confidence interval; FPM, flexible parametric model; HR, hazard ratio; LMWH, low molecular-weight heparin; OR, odds ratio.

^a^ Weight (kg)/height (m)^2^.

^b^ In mechanical prophylaxis, “active” includes foot pump and calf compression whereas “passive” is thromboembolic disease (TED) stockings.

### Overall performance

The AIC values, degrees of freedom, and deviances (twice the negative likelihood) for the parametric models are shown in Table 3, where the FPM is preferred (lowest value) by the AIC. The RSF is not included in Tables 2 and 3 because it is a nonparametric approach and is not fitted via the maximum likelihood algorithm; hence AIC cannot be calculated.

**Table 3.** Model Fit Statistics for Different Parametric Models Using Data From the National Joint Registry for England, Wales, Northern Ireland, and the Isle of Man, 2003–2015

Model	TKR			UKR			PFR		
	Degrees of Freedom	Deviance	AIC	Degrees of Freedom	Deviance	AIC	Degrees of Freedom	Deviance	AIC
Exponential model	14	77,276	77,304	14	17,929	17,957	14	3,547	3,575
Weibull model	15	77,258	77,288	15	17,926	17,956	15	3,535	3,565
Log-logistic model	15	77,251	77,281	15	17,922	17,952	15	3,531	3,561
FPM	18	76,606	76,642	18	17,829	17,865	16	3,505	3,537

Abbreviations: AIC, Akaike information criterion; FPM, flexible parametric model; PFR, patellofemoral replacement; TKR, total knee replacement; UKR, unicondylar knee replacement.

**Figure 1.**
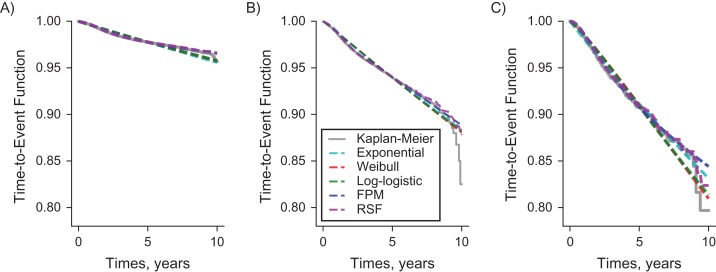
Observed and predicted probabilities of remaining event-free, using different models and data from the National Joint Registry for England, Wales, Northern Ireland, and the Isle of Man, 2003–2015. A) Total knee replacement; B) unicondylar knee replacement; C) patellofemoral replacement. Predicted probabilities of remaining event-free were obtained from different models: exponential model, Weibull model, log-logistic model, flexible parametric model (FPM), and random survival forest (RFS). The observed probability of remaining event-free was obtained from the Kaplan-Meier estimator.

**Figure 2.**
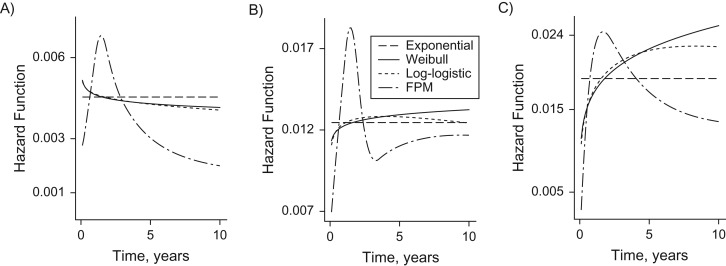
Hazard estimates for different parametric models, using data from the National Joint Registry for England, Wales, Northern Ireland, and the Isle of Man, 2003–2015. A) Total knee replacement; B) unicondylar knee replacement; C) patellofemoral replacement. FPM, flexible parametric model.

The averaged predicted survival curves over all individuals along with the observed (Kaplan-Meier) curve over time are plotted in Figure 1. The results show that the FPM and the RSF method captured the observed probabilities of remaining event-free accurately. The averaged hazard curves for the parametric models are also given in Figure 2, showing that the FPM can capture the increase and decrease of the hazard rate in the early and the later stages after primary surgery. This may explain its lower AIC values compared with the other parametric models. Figure 1 also suggests that there is insufficient information after year 8; thus only data up to this time point was used in subsequent analyses.

### Repeated *m*-fold cross-validation

Only the FPM and RSF approaches were considered for further comparison given their performance in the previous analysis. The integrated Brier score of the FPM and the RSF at 5 and 8 years are shown in Table 4. FPM and RSF yielded almost identical integrated Brier scores.

**Table 4.** Integrated Brier Score Using Data From the National Joint Registry for England, Wales, Northern Ireland, and the Isle of Man, 2003–2015

Model and Procedure	Integrated Brier Score			
	At 5 Years	95% CI	At 8 Years	95% CI
FPM				
TKR	0.014	0.014, 0.014	0.020	0.020, 0.020
UKR	0.036	0.036, 0.036	0.052	0.052, 0.052
PFR	0.058	0.058, 0.059	0.074	0.073, 0.075
RSF				
TKR	0.015	0.015, 0.015	0.020	0.020, 0.020
UKR	0.037	0.037, 0.037	0.052	0.052, 0.052
PFR	0.059	0.059, 0.059	0.073	0.072, 0.074

Abbreviations: CI, confidence interval; FPM, flexible parametric model; PFR, patellofemoral replacement; RSF, random survival forest; TKR, total knee replacement; UKR, unicondylar knee replacement.

The *C* indexes of the FPM and the RSF at 8 years are presented in Table 5. The FPM model had a higher *C* index across all procedures, with the greatest contrast versus the RSF models being for TKR, followed by UKR.

**Table 5.** *C* Index at 8 Years Using Data From the National Joint Registry for England, Wales, Northern Ireland, and the Isle of Man, 2003–2015

Model	TKR		UKR		PFR	
	*C* Index	95% CI	*C* Index	95% CI	*C* Index	95% CI
FPM	0.705	0.702, 0.707	0.639	0.634, 0.643	0.589	0.586, 0.592
RSF	0.660	0.655, 0.666	0.616	0.610, 0.621	0.579	0.575, 0.582

Abbreviations: CI, confidence interval; FPM, flexible parametric model; PFR, patellofemoral replacement; RSF, random survival forest; TKR, total knee replacement; UKR, unicondylar knee replacement.

**Figure 3.**
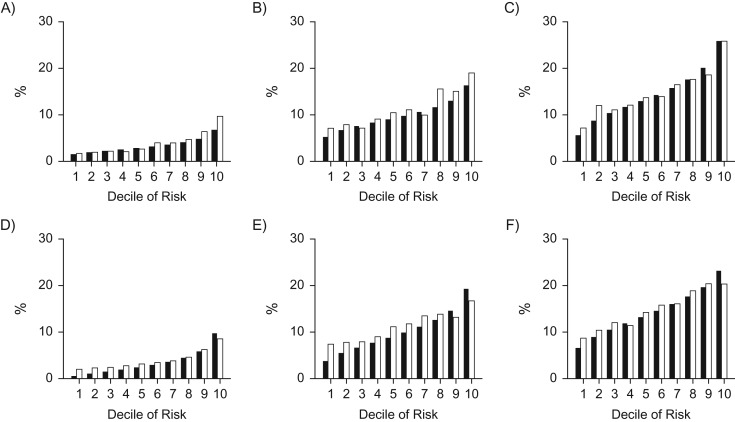
Calibration plots of prosthesis revision showing predicted risks (black bars) and observed risks (white bars) for different risk groups, using data from the National Joint Registry for England, Wales, Northern Ireland, and the Isle of Man, 2003–2015. A) Total knee replacement, results from the flexible parametric model; B) unicondylar knee replacement, results from the flexible parametric model; C) patellofemoral replacement, results from the flexible parametric model; D) total knee replacement, results from the random survival forest; E) unicondylar knee replacement, results from the random survival forest; F) patellofemoral replacement, results from the random survival forest.

Calibration was assessed by dividing the data into deciles of predicted risk of experiencing prosthesis revision within 8 years. Calibration plots were then constructed (Figure 3) to compare observed and average predicted risks for each decile. The absolute probabilities of prosthesis revision along with observed-to-predicted ratios of each decile for different models are also presented in Table 6.

**Table 6.** Observed Versus Predicted Risks of Prosthesis Revision for Different Risk Groups Using Data From the National Joint Registry for England, Wales, Northern Ireland, and the Isle of Man, 2003–2015

Model and Risk Decile	TKR		UKR		PFR	
	Predicted Probability, Mean (SD)^a^	Ratio of Observed to Predicted	Predicted Probability, Mean (SD)^a^	Ratio of Observed to Predicted	Predicted Probability, Mean (SD)^a^	Ratio of Observed to Predicted
FPM						
1	1.47 (0.0006)	1.16	5.33 (0.0106)	1.32	5.67 (0.0581)	1.28
2	1.89 (0.0005)	1.04	6.79 (0.0073)	1.18	8.83 (0.0483)	1.37
3	2.19 (0.0005)	1.00	7.67 (0.0070)	0.96	10.47 (0.0511)	1.05
4	2.48 (0.0005)	0.84	8.41 (0.0065)	1.02	11.77 (0.0453)	1.05
5	2.79 (0.0005)	0.97	9.11 (0.0055)	1.16	13.02 (0.0409)	1.01
6	3.14 (0.0006)	1.24	9.85 (0.0066)	1.14	14.35 (0.0436)	0.98
7	3.53 (0.0005)	1.12	10.70 (0.0077)	0.92	15.84 (0.0406)	1.04
8	4.04 (0.0008)	1.16	11.72 (0.0081)	1.34	17.67 (0.0562)	1.01
9	4.77 (0.0008)	1.36	13.1 (0.0116)	1.13	20.18 (0.0701)	0.92
10	6.71 (0.0017)	1.44	16.41 (0.0245)	1.16	25.99 (0.1186)	0.98
RSF						
1	0.64 (0.0041)	3.13	4.00 (0.0325)	1.84	6.70 (0.1375)	1.25
2	1.16 (0.0056)	1.97	5.71 (0.0272)	1.38	9.05 (0.1053)	1.22
3	1.59 (0.0071)	1.56	6.82 (0.0243)	1.18	10.59 (0.1141)	1.11
4	2.02 (0.0087)	1.35	7.84 (0.0265)	1.13	11.96 (0.1249)	1.09
5	2.49 (0.0116)	1.28	8.88 (0.0253)	1.13	13.26 (0.1231)	1.02
6	3.03 (0.0123)	1.13	10.01 (0.0287)	1.19	14.62 (0.1146)	1.00
7	3.68 (0.0131)	1.04	11.23 (0.0358)	1.23	16.09 (0.1194)	1.03
8	4.56 (0.0168)	1.03	12.64 (0.0420)	1.13	17.72 (0.1517)	1.00
9	5.93 (0.0271)	1.05	14.52 (0.0470)	0.88	19.69 (0.1695)	1.05
10	9.83 (0.0745)	0.87	19.09 (0.0700)	0.90	23.20 (0.2855)	0.90

Abbreviations: FPM, flexible parametric model; PFR, patellofemoral replacement; RSF, random survival forest; SD, standard deviation; TKR, total knee replacement; UKR, unicondylar knee replacement.

^a^ Predicted probabilities (%) are expressed as mean (SD).

The observed-to-predicted ratios indicate that the models tended to underestimate the risk in majority of cases. This underestimation may suggest that additional factors associated with revision are absent from the data set. However, the observation that RSF both underestimates the risks in the low-risk groups and overestimates the risk in the highest-risk decile suggests an overfitting bias despite the ensemble averaging over all trees.

We present additional analyses using 50 repeats of stratified 5-fold cross-validation in Web Tables 4 and 5; the results are similar to those in this section.

## DISCUSSION

Here we have presented a comparative evaluation of alternative survivorship models for knee replacement using the world’s largest knee-replacement clinical data set. A variety of performance metrics were used to evaluate the generated models. The flexible parametric survival model outperformed other methods although its predictive ability was, at best, modest. The FPM and RSF gave identical integrated Brier scores; however, FPM had a higher *C* index. The observed-to-predicted ratios indicated that both models tended to underestimate the risks in majority of risk groups.

Brier scores close to zero indicate that models are able to calculate underlying risks by usefully extracting information from data. The *C* index uses individual predicted probabilities to distinguish unrevised from revised cases, and our *C* index results show that the models are capable of providing meaningful individual predictions, with a range from 0.59 to 0.71 depending on the model chosen.

The main disadvantage of parametric methods is that the assumed underlying distribution may be misspecified. The FPM incorporates a parametric distribution with flexible complexity to minimize the problem of model misspecification. However, there is no theoretical basis for the number and locations of the knots for the estimation of the baseline scale (28). Other popular flexible methods include piecewise exponential models (53), Bayesian survival models (54), and alternative spline-based approaches (55). The RSF algorithm does not make any modeling assumptions and can handle nonlinear effects and interactions. However, categorization using data-dependent splits gives a suboptimal representation of a continuous variable (56), and the optimal setting of tuning parameters such as the number of trees, the splitting rule, and the number of randomly selected variables for each node split may also represent challenges with this method. Alternative machine learning techniques in modeling time-to-event data are Survival-SVM (57) and other ensemble schemes such as boosting methods (58).

We carried out a complete-case analysis assuming that data were missing at random and thus used only patients with complete data on the covariates of interest. Approximately 41.8% of data were excluded, most due to missing BMI data (40.2%). We consider that this is unlikely to affect the results of our comparative study, but this could be addressed using multiple imputation techniques (59, 60). However, results from previous studies using imputed BMI have produced results almost identical to those of selective complete-case analysis (21). The constructed models do not consider the competing risk of death, thus possibly biasing estimates of the prosthesis revision probability. These models can be further extended to accommodate competing risks in the calculation of the absolute risk for each individual (61, 62). Here we assumed a proportional-hazards spline model where time-dependent effects were not considered. This may also have caused bias in the risk estimates (63) of prosthesis revision. The flexible model can be further extended for possible improvement in fit by adding terms for interactions between covariates and the effect of time (27). Finally, an external validation to assess the generalizability and transportability of the model among different populations is required (64).

We created different algorithms to model time to event for the 3 knee replacement procedures because the demographic characteristics of the patient populations undergoing each, while overlapping, are distinct, and our aim was to model individual time-to-event estimates based on real-world data. However, the observed differences in revision events between the procedure types raises the separate question of whether this differential revision rate is a function of the procedure, the prosthesis, the patient, or a combination. One approach to model this would be to select random data sets from the overlapping variable characteristics within the cohorts and to estimate a joint model with indicator variables for the different procedures. An alternative modeling approach, such as propensity score matching, might also be employed. However, with both approaches the residual challenge of unobserved confounding would remain (65).

Our findings indicate that predictive algorithms based upon the largest current knee replacement and surgical outcomes data set have a modest ability to predict individual survival performance. Further variables not captured within routinely collected clinical audit data sets, such as time between prosthesis insertion and diagnosis of failure rather than time to revision surgery, and the development of novel algorithm methodologies may enhance predictive ability in the future. However, use of current data-driven point estimates of prosthesis performance despite modest discriminatory ability may still be sufficient to help inform preference-based decision-making, although clinical trials of their implementation will be required to confirm their utility.

## Supplementary Material

Web Material

## Abbreviations

AIC: Akaike information criterion
BMI: body mass index
FPM: flexible parametric model
NJR: National Joint Registry for England, Wales, Northern Ireland, and the Isle of Man
PFR: patellofemoral replacement
RSF: random survival forest
TKR: total knee replacement
UKR: unicondylar knee replacement
