# Supplementary material for: Estimating an Individual’s Probability of Revision Surgery After Knee Replacement: A Comparison of Modeling Approaches Using a National Data Set
Source: Am J Epidemiol. 2018 Jun 11;187(10):2252–62. doi: 10.1093/aje/kwy121 (PMC6166214; doi:10.1093/aje/kwy121)
Supplement: Web Material [file kwy121_wilkinson_web_material_final.pdf]

Web Material

Estimating an Individual's Probability of Revision Surgery After Knee Replacement: A Comparison of Modeling Approaches Using a National Data Set

Parham Aram, Lea Trela-Larsen, Adrian Sayers, Andrew F. Hills, Ashley W. Blom, Eugene V. McCloskey, Visakan Kadirkamanathan, and Jeremy M. Wilkinson

Web Table 1. Fractional Polynomial Models for the Relationship Between Age and BMI and Prosthesis Failure Using Data From the National Joint Registry for England, Wales, Northern Ireland and the Isle of Man (Between April 2003 and September 2015).

Procedure and Variable	Deviance Difference		Powers (P value)	
	m=1[a] versus linear	m=1 versus m=2[b]	m=1	m=2
TKR				
Age	1.53	2.91	0.5 (0.22)	-2,0 (0.23)
BMI	1.72	1.97	0.5 (0.19)	-2,-2 (0.37)
UKR				
Age	1.19	0.25	0 (0.27)	-2,-2 (0.88)
BMI	0.03	0.08	-1 (0.86)	-2,-2 (0.96)
PFR				
Age	2.31	3.86	3 (0.13)	0,3 (0.14)
BMI	0.77	0.36	-1 (0.38)	0,0.5 (0.83)

Abbreviations: PFR, Patellofemoral Replacement; TKR, Total Knee Replacement;UKR, Unicondylar Knee Replacement.

[a] m=1 denotes first degree fractional polynomials.

[b] m=2 denotes second degree fractional polynomials.

Web Table 2. Parametric and Semi-parametric Cox Models of Prosthesis Survivorship for Unicondylar Knee Replacement Using Data From the National Joint Registry for England, Wales, Northern Ireland and the Isle of Man (Between April 2003 and September 2015).

	Exponential Model		Weibull Model		FPM		Cox Model		Log-logistic Model	
Characteristic	HR	95% CI	HR	95% CI	HR	95% CI	HR	95% CI	OR	95% CI
Age, years	0.972	0.967, 0.977	0.973	0.968, 0.978	0.9722	0.967-0.977	0.972	0.967, 0.977	0.972	0.967, 0.978
BMI[a]	1.004	0.994, 1.014	1.004	0.994, 1.014	1.004	0.994, 1.014	1.004	0.994, 1.014	1.004	0.994, 1.014
Gender										
Female	1.000	Referent	1.000	Referent	1.000	Referent	1.000	Referent	1.000	Referent
Male	0.976	0.887, 1.074	0.978	0.891, 1.073	0.974	0.885, 1.073	0.975	0.886, 1.073	0.980	0.892, 1.077
ASA Physical Status										
P2	1.000	Referent	1.000	Referent	1.000	Referent	1.000	Referent	1.000	Referent
P1	0.966	0.857, 1.088	0.966	0.861, 1.084	0.966	0.858, 1.088	0.965	0.857, 1.087	0.966	0.860-1.086
P3	1.194	1.420, 1.003	1.186	1.003, 1.404	1.193	1.002, 1.419	1.194	1.003, 1.420	1.190	1.003, 1.412
Chemical Prophylaxis										
LMWH± Aspirin	1.000	Referent	1.000	Referent	1.000	Referent	1.000	Referent	1.000	Referent
Aspirin Only	0.918	0.797, 1.056	0.913	0.797, 1.046	0.935	0.812, 1.077	0.941	0.817, 1.085	0.919	0.800, 1.056
None	1.058	0.905, 1.237	1.045	0.898, 1.216	1.076	0.920, 1.259	1.079	0.922, 1.263	1.054	0.902, 1.231
Other/Other Combinations	0.903	0.787, 1.037	0.915	0.800, 1.046	0.886	0.787, 1.037	0.878	0.764, 1.008	0.911	0.796, 1.043
Mechanical Prophylaxisa[b]										
Both	1.000	Referent	1.000	Referent	1.000	Referent	1.000	Referent	1.000	Referent
Active	0.910	0.796, 1.041	0.914	0.803, 1.041	0.909	0.795, 1.039	0.909	0.795, 1.039	0.915	0.802, 1.043
Passive	0.955	0.851, 1.072	0.954	0.853, 1.066	0.957	0.853, 1.074	0.959	0.854, 1.076	0.950	0.848, 1.064
None	1.354	1.106, 1.657	1.328	1.090, 1.616	1.374	1.122, 1.683	1.366	1.115, 1.674	1.343	1.096, 1.646
Other/Other Combinations	1.281	0.802, 2.047	1.273	0.809, 2.003	1.276	0.799, 2.039	1.277	0.800, 2.041	1.256	0.792, 1.994
Operation Type										
Unilateral	1.000	Referent	1.000	Referent	1.000	Referent	1.000	Referent	1.000	Referent
Simultaneous Bilateral	0.570	0.442, 0.735	0.579	0.453, 0.742	0.572	0.443, 0.738	0.571	0.442, 0.736	0.577	0.450, 0.738

Abbreviations: ASA, American Society of Anesthesiologists; BMI, Body Mass Index; CI, Confidence Interval; FPM, Flexible Parametric Model; HR, Hazard Ratio; OR, Odds Ratio.

[a] Weight (kg)/height $(m)^2$.

[b] In Mechanical Prophylaxis , Active includes foot pump and calf compression whereas Passive is ThromboEmbolic Disease (TED) stockings.

Web Table 3. Parametric and Semi-parametric Cox Models of Prosthesis Survivorship for Patellofemoral Replacement Using Data From the National Joint Registry for England, Wales, Northern Ireland and the Isle of Man (Between April 2003 and September 2015).

	Exponential Model		Weibull Model		FPM		Cox Model		Log-logistic Model	
Characteristic	HR	95% CI	HR	95% CI	HR	95% CI	HR	95% CI	OR	95% CI
Age, years	0.988	0.978, 0.998	0.990	0.981, 0.998	0.988	0.978, 0.998	0.988	0.978, 0.998	0.989	0.981, 0.998
BMI[a]	1.024	1.004, 1.044	1.020	1.003, 1.038	1.024	1.004, 1.045	1.024	1.004, 1.045	1.021	1.003, 1.039
Gender										
Female	1.000	Referent	1.000	Referent	1.000	Referent	1.000	Referent	1.000	Referent
Male	1.492	1.186, 1.879	1.414	1.160, 1.723	1.493	1.186, 1.879	1.494	1.187, 1.880	1.433	1.168, 1.757
ASA Physical Status										
P2	1.000	Referent	1.000	Referent	1.000	Referent	1.000	Referent	1.000	Referent
P1	0.924	0.719, 1.187	0.928	0.750, 1.149	0.923	0.718, 1.185	0.919	0.715, 1.181	0.922	0.741, 1.147
P3	0.965	0.652, 1.426	0.964	0.691, 1.346	0.967	0.654, 1.430	0.964	0.652, 1.425	0.981	0.698, 1.380
Chemical Prophylaxis										
LMWH± Aspirin	1.000	Referent	1.000	Referent	1.000	Referent	1.000	Referent	1.000	Referent
Aspirin Only	0.855	0.634, 1.152	0.844	0.654, 1.088	0.864	0.640, 1.166	0.854	0.632, 1.154	0.835	0.643,1.083
None	1.264	0.920, 1.738	1.164	0.855, 1.531	1.274	0.925, 1.756	1.276	0.926, 1.759	1.184	0.889, 1.576
Other/Other Combinations	0.784	0.574, 1.070	0.839	0.642, 1.097	0.778	0.569, 1.063	0.773	0.566, 1.057	0.833	0.636, 1.089
Mechanical Prophylaxis[b]										
Both	1.000	Referent	1.000	Referent	1.000	Referent	1.000	Referent	1.000	Referent
Active	0.762	0.563, 1.032	0.794	0.613, 1.028	0.761	0.562, 1.031	0.764	0.564, 1.034	0.793	0.610, 1.030
Passive	1.233	0.965, 1.575	1.182	0.958, 1.458	1.230	0.962, 1.572	1.232	0.964, 1.575	1.176	0.949, 1.458
None	1.541	1.046, 2.271	1.402	1.004, 1.958	1.547	1.049, 2.282	1.547	1.048, 2.281	1.435	1.011, 2.036
Other/Other Combinations	0.714	0.292, 1.747	0.735	0.343, 1.576	0.712	0.291, 1.742	0.708	0.289, 1.733	0.727	0.339, 1.559
Operation Type										
Unilateral	1.000	Referent	1.000	Referent	1.000	Referent	1.000	Referent	1.000	Referent
Simultaneous Bilateral	0.425	0.257, 0.704	0.484	0.309, 0.734	0.432	0.256, 0.700	0.428	0.259, 0.709	0.478	0.311, 0.734

Abbreviations: ASA, American Society of Anesthesiologists; BMI, Body Mass Index; CI, Confidence Interval; FPM, Flexible Parametric Model; HR, Hazard Ratio; OR, Odds Ratio.

[a] Weight (kg)/height (m)^2 .

[b] In mechanical prophylaxis , Active includes foot pump and calf compression whereas Passive is ThromboEmbolic Disease (TED) stockings.

Web Table 4. Results of Brier Score and C Index for 8 Years Survival Using Data From the National Joint Registry for England, Wales, Northern Ireland and the Isle of Man (Between April 2003 and September 2015).

Model and Procedure	Integrated Brier Score	95% CI	C index	95% CI
FPM				
TKR	0.020	0.020, 0.020	0.701	0.698, 0.704
UKR	0.052	0.052, 0.052	0.632	0.627, 0.637
PFR	0.073	0.073, 0.074	0.588	0.586, 0.591
RSF				
TKR	0.020	0.020, 0.020	0.656	0.652, 0.661
UKR	0.052	0.052, 0.052	0.602	0.596, 0.608
PFR	0.073	0.073, 0.074	0.578	0.575, 0.582

Abbreviations: C Index, Concordance Index; CI, Confidence Interval; FPM, Flexible Parametric Model; PFR, Patellofemoral Replacement; RSF, Random Survival Forest; TKR, Total Knee Replacement; UKR, Unicondylar Knee Replacement.

Web Table 5. Observed Versus Predicted Risks of Prosthesis Revision for Different Risk Groups Using Data From the National Joint Registry for England, Wales, Northern Ireland and the Isle of Man (Between April 2003 and September 2015).

Model and Risk Group	TKR		UKR		PFR	
	Predicted Probability, mean (SD)[a]	Ratio of Observed to Predicted	Predicted Probability, mean (SD)	Ratio of Observed to Predicted	Predicted Probability, mean (SD)	Ratio of Observed to Predicted
FPM						
1	1.47 (0.0006)	1.16	5.34 (0.0095)	1.33	5.69 (0.0489)	1.26
2	1.89 (0.0005)	1.04	6.80 (0.0076)	1.16	8.83 (0.0581)	1.36
3	2.19 (0.0006)	1.00	7.67 (0.0066)	0.93	10.47 (0.0511)	1.06
4	2.48 (0.0006)	0.84	8.41 (0.0061)	1.08	11.78 (0.0368)	1.03
5	2.79 (0.0005)	0.95	9.11 (0.0059)	1.15	13.03 (0.0392)	1.05
6	3.14 (0.0006)	1.27	9.85 (0.0062)	1.13	14.35 (0.0407)	0.97
7	3.53 (0.0006)	1.12	10.70 (0.0080)	0.93	15.85 (0.0346)	1.04
8	4.04 (0.0006)	1.17	11.72 (0.0091)	1.33	17.68 (0.0468)	1.00
9	4.77 (0.0010)	1.34	13.10 (0.0099)	1.15	20.18 (0.0509)	0.92
10	6.71 (0.0016)	1.44	16.41 (0.0197)	1.16	25.95 (0.0996)	1.00
RSF						
1	0.64 (0.0047)	3.15	3.85 (0.0469)	1.92	6.67 (0.1305)	1.30
2	1.16 (0.0044)	1.98	5.59 (0.0440)	1.4	9.02 (0.136)	1.15
3	1.59 (0.0053)	1.53	6.74 (0.0482)	1.18	10.59 (0.1464)	1.14
4	2.02 (0.0063)	1.37	7.78 (0.0457)	1.16	11.97 (0.1497)	0.95
5	2.49 (0.0082)	1.27	8.84 (0.0477)	1.26	13.29 (0.1524)	1.07
6	3.03 (0.0081)	1.14	9.98 (0.0475)	1.18	14.66 (0.1391)	1.08
7	3.68 (0.0092)	1.04	11.23 (0.0482)	1.20	16.10 (0.1435)	1.00
8	4.56 (0.0134)	1.01	12.70 (0.0680)	1.09	17.73 (0.1704)	1.06
9	5.93 (0.0195)	1.05	14.67 (0.0954)	0.90	19.72 (0.227)	1.03
10	9.83 (0.0493)	0.87	19.38 (0.1456)	0.86	23.26 (0.3181)	0.87

Abbreviations: FPM, Flexible Parametric Model; PFR, Patellofemoral Replacement; RSF, Random Survival Forest; SD, Standard Deviation; TKR, Total Knee Replacement; UKR, Unicondylar Knee Replacement.

[a] Predicted probabilities (%) are expressed as mean (standard deviation)
